# Sweat monitoring beneath garments using passive, wireless resonant sensors interfaced with laser-ablated microfluidics

**DOI:** 10.1038/s41746-020-0270-2

**Published:** 2020-04-30

**Authors:** Adam R. Carr, Yash H. Patel, Charles R. Neff, Sadaf Charkhabi, Nathaniel E. Kallmyer, Hector F. Angus, Nigel F. Reuel

**Affiliations:** 1 Department of Chemical and Biological Engineering, Iowa State University, Ames, IA USA; 2 Department of Kinesiology, Iowa State University, Ames, IA USA

**Keywords:** Chemical engineering, Preclinical research

## Abstract

Sweat loss can help determine hydration status of individuals working in harsh conditions, which is especially relevant to those who wear thick personal protective equipment (PPE) such as firefighters. A wireless, passive, conformable sweat sensor sticker is described here that can be worn under and interrogated through thick clothing to simultaneously measure sweat loss volume and conductivity. The sticker consists of a laser-ablated, microfluidic channel and a resonant sensor transducer. The resonant sensor is wirelessly read with a handheld vector network analyzer coupled to two, co-planar, interrogation antennas that measure the transmission loss. A sweat proxy is used to fill the channels and it is determined that the sensor can orthogonally determine the sweat conductivity and volume filled in the channel via peak transmission loss magnitude and frequency respectively. A four-person study is then used to determine level of sensor variance caused by local tissue dielectric heterogeneity and sensor-reader orientation.

## Introduction

The development of sweat analytical devices has been an active area of research in recent years^1,2^. Sweat is rich in biomarkers with clinical relevance and can be sampled non-invasively^3–5^. There is also a putative link between sweat sodium (Na + ), potassium (K + ), and chloride (Cl-) levels, and sweat rate and hydration status. Besides biomarkers, sweat rate, total loss, and local sweat loss have potential uses for monitoring hydration status^6–8^. Monitoring sweat loss specifically finds applications in sports performance monitoring^7,9–11^ and general health monitoring^12–14^. This can be especially beneficial for individuals working in harsh environments such as construction workers working in hot, humid climates or firefighters in structural fires. Firefighters, in particular, are at a high risk of dehydration. Studies have shown that due to the harsh conditions in a structural fire even ad libitum drinking may be insufficient to properly rehydrate^15,16^. Devices that could detect on-body measurement of sweat loss could help prevent serious health complications from dehydration in firefighters and help emergency medical technician personnel know if additional measures should take place beyond intake of fluids for proper rehydration. However, potential issues with measuring changes in body mass are (1) hidden water mass absorbed into clothing and (2) pooled sweat on the skin surface. Other considerations to factor are the effects of fitness level, body type, and heat acclimation on sweat rates^6,17,18^. Also, variation in sweating rates between individuals and regions of the body need to be accounted for in any sweat loss analysis^19,20^ with some possible correlations between regions^21^. To realize robust sweat monitoring, tailored sensors capable of multi-region monitoring are needed. Low-cost sweat rate and sweat composition sensors need to be developed, especially ones that can be worn comfortably below personal protective equipment (PPE).

Sweat sensors already presented in the literature can be classified by two distinct features: sweat sampling method and interrogation method. Example sample handling strategies include use of microfluidics^10,22–25^, wicking materials^26–29^, and natural ventilation strategies^30,31^. The most accurate method is natural ventilation as it can detect both sensible (liquid) and insensible (vapor) sweat loss^31^, but wearable implementation can prove to be difficult and bulky particularly under PPE. Wicking materials and microfluidics are simpler for wearable implementation with microfluidics having the advantage for reusable sensors whereas wicking materials are usually single-use devices. Transduction methods include potentiometry, capacitance, optical, and impedance. These transduction methods have been implemented with great success thanks to miniaturized electronics and ability to wireless interrogate signals via Wi-Fi, Bluetooth, and near field connections. Even with the recent advances in sweat sensor development one application that proves difficult to implement is undercoat perspiration. This is due to either the transduction method requiring a direct line of sight (e.g., optical), or bulky sensor implementation with multiple powered parts and integrated circuit boards. These become especially problematic for applications where the user is already encumbered with bulky gear and thick PPE, such as firefighters.

One sensor type that could decrease the size of wireless sweat rate sensors for applications through thick PPE is resonant sensors^23^. As their name denotes, these are simple circuits that resonate at a specific frequency dependent on their effective inductance and capacitance. Changes in permittivity of the surrounding medium can affect the latter parameter, and thus shift the resonant frequency^32^. Wireless resonant sensors have been used for several biomedical applications in the past including intraocular^33^, wireless power transfer for implanted sensor^34–36^, and vital signs^37,38^. This class of sensors has also been demonstrated to monitor biofluids for monitoring of proteins^12,39^ and also have the potential to characterize tissue dielectric properties^40^ and enzyme activity^41^. Resonant sensors have the potential to be a simple solution to sweat analysis.

**Fig. 1:**
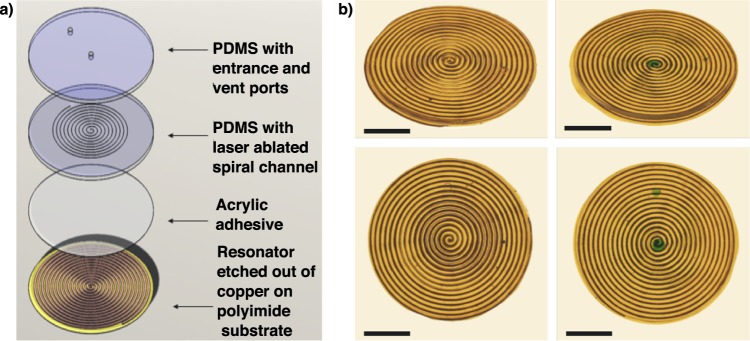
Resonant sensor for sweat rate and composition. **a** Exploded view of resonant sweat sensor with layers labeled. **b** Photographs from angled and top view of sensor both empty (on the left) and filled with dyed DI water (on the right), scale bar = 10 mm.

**Fig. 2:**
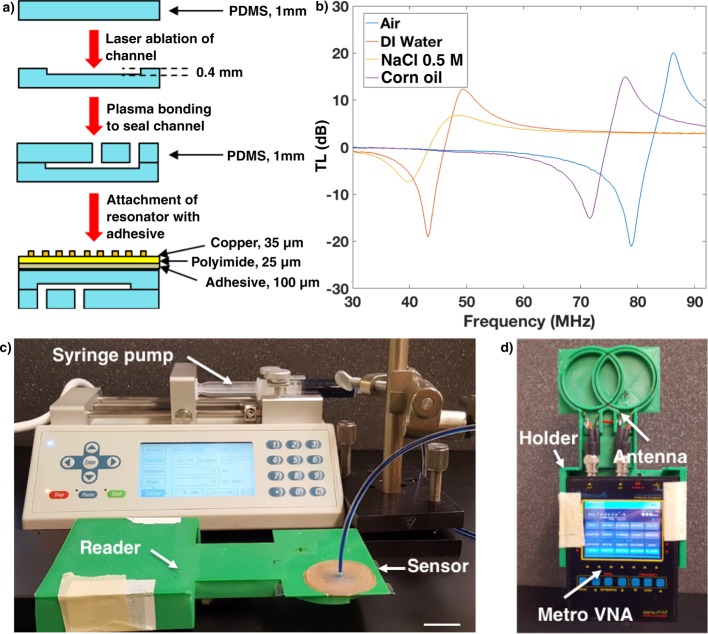
Sensor fabrication and interrogation. **a** Flow diagram for sensor fabrication with side view showing layer build up. **b** Bode plot showing the effect of different dielectric media on the resonator’s transmission loss. Resonator was attached to bottom of petri-dish with 10 mL of liquid for all trials. **c** Experimental setup for characterization of sensor using a syringe pump to fill channels with a custom reader, scale bar = 2 cm. **d** Custom reader setup including 3D printed holder, double loop interrogation antenna, and portable VNA (MetroVNA).

This study reports the fabrication of a resonant sticker that can analyze sweat rate and sweat conductivity, which correlates to overall ion concentration. The sticker sensor is composed of fluid handling channels (PDMS) with an adhered resonator (Fig. 1). The resonator is in the shape of an Archimedean spiral, and is chemically etched out of a copper coated polyimide substrate (DuPont Pyralux) with an indelible marker mask. The channels are also in the shape of an Archimedean spiral and fabricated in polydimethylsiloxane (PDMS) using a craft laser cutter and sealed using a separate piece of PDMS by plasma bonding (Fig. 2a). An Archimedean spiral was chosen for the geometry of the resonator as it works well for inductively coupled sensing systems, is simple to fabricate, and has been characterized previously in our lab^42^. The vector network analyzer (VNA) analyzes the transmission loss (TL) of the reader antenna/resonator system, which changes in amplitude and peak frequency (*f*_o_) and is determined by the complex permittivity of the surrounding medium (Fig. 2b). For this study, the sweat sensor sticker was analyzed both on the reader and through thick firefighter PPE (Morning Pride TAILS) to calibrate the sensor response and determine ability to read through clothing (see Supplementary Fig. 1). The sensor was analyzed using the custom reader and filled using a syringe pump (Fig. 2c). The custom reader was fabricated out of a low-cost, portable vector network analyzer (VNA, MetroVNA) and fitted into a custom holder with a double coil antenna (Fig. 2d) used to interrogate the sweat sticker via inductive coupling between a reader antenna and resonant sensor. We also measured the sensor on a small cohort of human subjects to determine sources of variability that we will need to control before attempting a larger human trial. Finally, we address the current limitations and detail next steps to enable resonant sensors for sweat analysis.

## Results

The sweat analysis sticker developed for this study is composed of microfluidic channels to distribute the sample across the resonator, which can then wirelessly and rapidly transduce local sweat loss and sweat conductivity. First, we will discuss the design and fabrication of bespoke microfluidic devices that can be rapidly produced to accommodate different sweat volumes (as each individual has different sweat levels). Second we will detail the design and response of the resonant sensors to transduce the conductivity (ion concentration) and overall sweat volume. These include correlations through PPE. Finally, we will study the effect of on-body resonator response and detail considerations that will have to go into a realized design for wearable sweat resonant sensors.

### Laser ablation

Microfluidics are most frequently fabricated using photo- and soft lithographic methods, which utilize a master mold to fabricate microchannels and structures in elastomeric materials. This technique is good for rapid replication of a single microfluidic design, but can become cumbersome during the prototyping stage where a new master mold has to be made for every design change. Another method to rapidly fabricate microfluidic devices is laser ablation of polymer blocks^43^. Although the literature has reported laser ablation of PDMS to make channels^44^, we were not able to find a convenient model to describe how the depth and width of channels in PDMS are controlled by the power and duration of a stock CO_2_ laser cutter. Applying a similar approach found in literature^45^ and with the aid of a 3D digital microscope, we developed a model for laser-ablated channel dimension. Eighty-eight straight channels were ablated into pre-cured sheets of PDMS and the resulting dimensions were determined using an Olympus DSX110 digital microscope (see Supplementary Fig. 2).

The depth model, fit to the analyzed data, was derived from a mass balance where the mass flux, m” is a function of the heat flux from the laser, *Q*”, and the heat of pyrolysis of the material, *L*,

1 $$m{\prime\prime} = \left( {Q{\prime\prime} - Q\prime\prime _o} \right)/L$$

where *Q*”_o_ represents the critical heat flux when pyrolysis of material will begin. The heat flux from the laser, *Q*” can be represented as

2 $$Q\prime\prime = \alpha \varphi _o\Delta t$$

where *α* is the absorptance of the material, *φ*_o_ is the laser power, and Δ*t* is the irradiance time, which is a function of half the laser length of the Gaussian beam width, a, and the cutting speed, *v*, Δ*t* = *a*/*v*. Specifying the ablated area to be *πa*^2^ and the density of PDMS (*ρ*), the depth (*D*) of the channel can then be determined as,

3 $$D = \frac{{c_1\varphi _o}}{v} - c_2$$

With constants$c_1 = \frac{\alpha }{{\rho \pi La}}$ and $c_2 = \frac{{Q\prime\prime _o}}{{\rho \pi La^{2^ \cdot }}}$.

Channels were ablated in PDMS using laser power settings between 8 and 24 W and cutting speed between 0.008 and 0.04 m/s using a low-cost, craft laser cutter in vector mode (GlowForge Plus). Resulting channel depth data were fit by the depth model (Eq. 3) and resulting model parameters of *c*_1_ = 0.5357 (±0.063 at 95% confidence interval) µm/(J/m) and *c*_2_ = 18.5 (±45.1) µm with coefficient of determination *R*^2^ = 0.943 and root-mean-squared error (RMSE) = 45.10 µm (Supplementary Fig. 2). A likely reason for the large RMSE value is the difficulty to cure PDMS to a uniform height. Even a small difference of 50 microns in height would have an effect on the focal point of the laser above the substrate, which would create larger than expected channels in some cases. On the other hand, the model showed that the laser ablation technique is adequate for channels with an aspect ratio >1.4 (minimum channel height of 100 µm with average width of 140 µm), which can accommodate volumes of sweat >14 µL, assuming a channel length of 1 m.

The width of the channels is a strong function of the laser beam diameter and remains relatively constant for different laser powers and cutting speeds. Upon inspection it was determined there is a linear correlation between channel width and channel depth (Eq. 4):

4 $$W = 0.073D + 110$$

where *W* is the width of the channel and RMSE of the model is 17 µm, with a correlation coefficient of 0.635. Although the effect of laser power and cutting speed on the channel widths and depths from the craft laser cutter has relatively large variation they were found suitable for design of devices in this study for sweat storage and handling. A major benefit of using laser ablation over other soft lithography or stereolithagraphy printing is the ability to rapidly prototype designs. This can be useful in designing different size channels to accommodate individuals with lower or higher sweating rates. Laser cutting PDMS coupled with plasma bonding results in a process, which can go from conceptual design to prototype in a short amount of time. As an example four designs (see Supplementary Fig. 2) were created and tested within 15 min in our lab.

### Effect of sweat conductivity on sensor

Sweat is a rich milieu of electrolytes, metabolites, and amino acids^3,46^; however, the dominant component for each person is sodium chloride (NaCl) making up most of the charge carriers in sweat and thus mostly responsible for sweat conductivity^47^. Although the conductivity of sweat is not an established clinical method to monitor hydration studies have demonstrated correlation between hydration level and sweat conductivity^48–50^. Because of the relatively large amount of NaCl present in sweat, simple solutions of NaCl have been used to mimic the conductivity of human sweat, rather than reconstituting all lower concentration contributors^2,47,49^. We used similar sweat proxy solutions to test the response of the flexible resonant sensor stickers, detecting changes in resonant frequency (*f*_o_) and relative power transmission loss (TL) to increased conductivity, e.g., NaCl concentration from 0.01 to 0.1 mol/L.

**Fig. 3:**
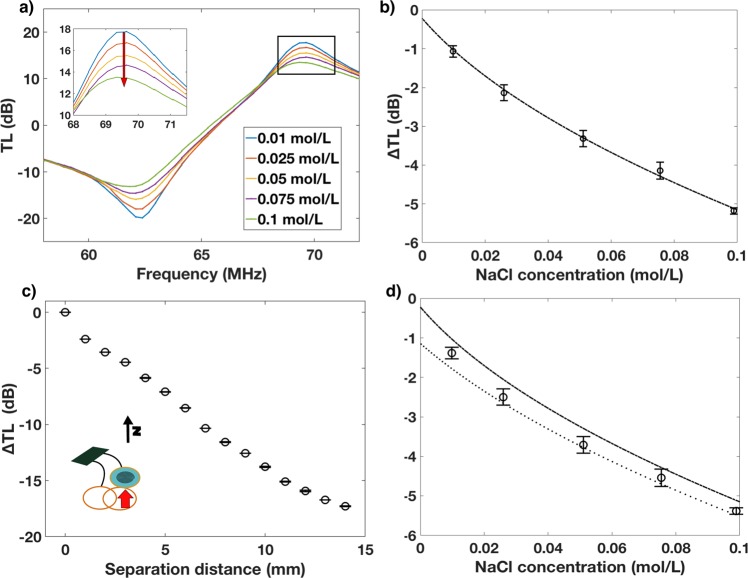
Effect of conductivity on ∆TL. **a** Bode plot TL for frequency sweep of sticker filled with solutions with increasing (red arrow) concentration of NaCl. **b** Quadratic model of ∆TL as a function of solution conductivity for resonator directly on reader antenna. **c** Effect of separation distance between resonator and reader on ∆TL. **d** Circles, test data of different conductivity solutions through PPE with model from **b**) (-.) and model with correction offset based off garment thickness(..); *n* > 20, error bars s. d.

From the TL data it is observed that the amplitude of the resonant peak decreases as the conductivity increases meaning that the losses decrease with increasing concentration (Fig. 3a). This is apparently in contrast with other reports, which show that losses increase with concentration due to the decrease in permittivity and increase in conductivity^51^. A possible explanation for this is that the microfluidic chip with filling solution is acting as a matching network for the resonator and reader antenna system. As the salt solution is increasing it is matching the impedance of the resonator to the reader antenna, leading to a more lossless power transfer. A similar phenomena was recently observed in a study conducted in our lab characterizing solvated ions using resonator arrays^41,42^.

The change in TL (∆TL) was calculated from the zero concentration solution and a quadratic model was fit to the data of the form

5 $$\Delta {\mathrm{TL}} = p_1\sigma ^2 + p_2\sigma$$

with *R*^2^ of 0.964 with *p*_1_ = 0.19 and *p*_2_ = –1.08 (Fig. 3b). NaCl solutions were then tested in the sensor while reading through the thick PPE to determine the efficacy of the model to predict conductivity through the material. There was a consistent offset between the model and the PPE data, which is purported to be a result of displacement between the reader antenna and the resonator due to the thickness of the PPE. In order to correct for this offset, the effect of separation distance on ∆TL was determined (Fig. 3c), which fit a linear model with a slope of –1.345 ∆TL/mm. With the PPE thickness being 0.9 mm the offset was calculated as –1.2 once this correction factor was added to the model we observed a better prediction of conductivity through PPE for conductivities about 0.01 mol/L, which is the starting point for range of sweat conductivity (Fig. 3d).

### Effect of sweat volume on sensor

**Fig. 4:**
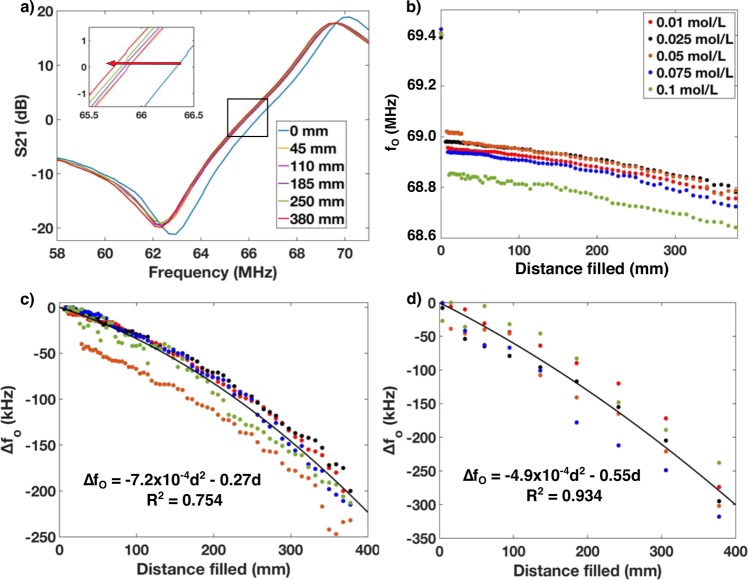
Effect of fluid filling on ∆*f*o. **a** Bode plot TL for frequency sweep of sticker with increasing (red arrow) distance filled in microfluidic channel. **b** Raw *f*_o_ data from empty channel to completely filled for different concentrations of NaCl. **c** Normalized data from filling channel directly on the reader antenna for different sweat conductivities with model fit (black line). **d** Normalized data from channel filled through PPE with model fit (black line).

The effect of sweat volume (distance filled in the sensor channel) on resonant response was also determined. The distance filled has a significant effect on the change in *f*_o_ (∆*f*_o_, Fig. 4a). We hypothesize that as the channel fills with sweat, the resonant frequency will shift down as the relative permittivity of water is ~80 times greater than that of air, thus increasing the capacitance in the channel as capacitance is directly proportional to relative permittivity. This inverse relationship of capacitance and resonant frequency is described by the fundamental equation for resonant frequency,

6 $$f_o = \frac{1}{{2\pi \sqrt {LC} }}$$

where *L* is the inductance and *C* is the capacitance.

When initially filling the channels (going from air to small amount of fluid) we observe a relatively large, initial step decrease in resonant frequency (Fig. 4b). After this initial step change a more gradual, parabolic decrease is observed upon addition of more fluid. For these experiments ∆*f*_o_ was calculated by normalizing to the first data point with fluid. The model for both the resonator on the reader and through PPE had the form of

7 $$\Delta f_o = p_1d^2 + p_2d$$

where *p*_1_ and *p*_2_ are constants and *d* is the distance filled in the channel. For the resonator directly on the reader *p*_1 _= –7.2 × 10^-4^ kHz/mm^2^ and *p*_2_ = –0.27 kHz/mm with *R*^2^ of 0.754, whereas for the resonator through PPE *p*_1_ = –4.9 × 10^–4^ kHz/mm^2^ and *p*_2_ = –0.55 kHz/mm and *R*^2^ of 0.934.

The quadratic response can be attributed to the fact that the sensor is observed to be more sensitive to dielectric changes towards the edge of the spiral than in the center (see Supplementary Fig. 3). As the sensor is initially filled from the center, the sensor gain is not as steep as when the sensor is filled towards the outer edge, thus resulting in a quadratic transfer function. This phenomena can also be explained by the fact that the inner turns of a planar spiral resonator contribute less-positive mutual inductance between the resonator and reader antenna and thus show a lower frequency response than that outer turns^52^.

Data sets for filling of the sensor either read directly on the reader and through the PPE both showed a quadratic response (Fig. 4c, d) although the PPE data shows a faster changing slope than that taken directly on the reader. This would mean that a calibration curve for sweat rate should be generated for different types of PPE. However, since the response is quadratic a simple three to four-point calibration would be sufficient. Also, if larger volume channels were used to accommodate a higher local sweat rate then the increase in fluid volume would decrease the resonant frequency even further with distance filled. It is important to also note that the effect of filling fluid on the ∆TL response for conductivity of the solution was found to be insignificant (see Supplementary Fig. 4). This is a significant finding for this simple sensor architecture as it indicates that conductivity and sweat volume can be measured orthogonally using the same resonator sticker without the need for a multiplexed sensing system.

The effect of bending on the channel cross sectional area and high-frequency stress cycles on the sensors response were tested (see Supplementary Fig. 5). The cross sectional area did not appreciably change with bending meaning that the curvature of the body will have little effect on sensor performance. Cycle fatigue testing was also performed to determine the effect of repeated elastic deformation on sensor performance. From these tests it was determined that the sensor response remained stable even after 2000 cycles.

### Considerations for on-body deployment

The ultimate goal for these sensors is to be deployed as inexpensive, single-use stickers for measuring personal sweat rate and sweat composition. The fabrication of these sensors (etched or screen printed) make for an inexpensive wireless solution compared to other wireless transducers that involve more complex PCB boards, integrated circuits, and assembly. However, a few drawbacks to these simple resonators are their sensitivity to any dielectric material within ~1.5 cm proximity (which also affect the fringing fields), as well as sensitivity to position and orientation between reader and sensor, which affects the sensor response. Although other forms of resonator sensors have been performed on human subjects before^53,54^, the orientational dependence of the TL signal between the portable reader and an Archimedean spiral resonator needed to be determined for the implementation of these style of resonators in wearable applications.

**Fig. 5:**
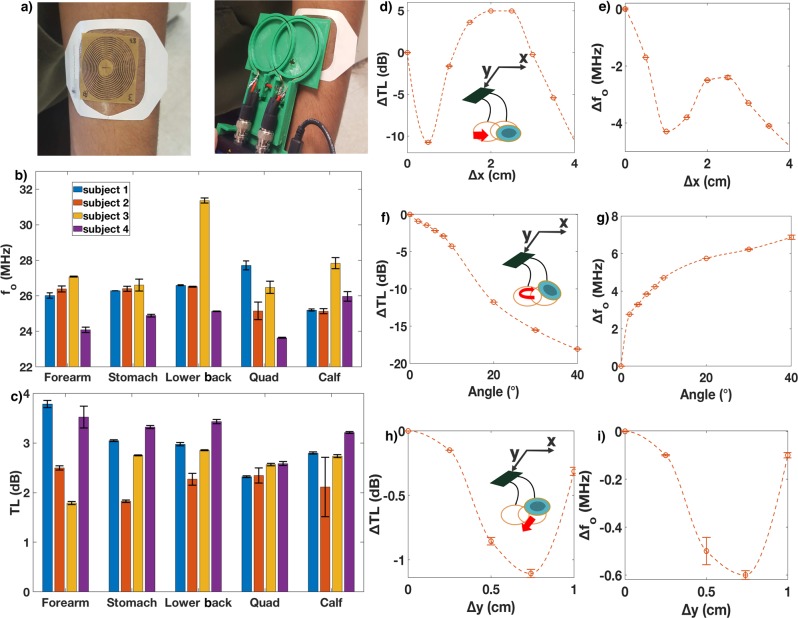
Orientational dependence and sensor wearablility. **a** Resonant sensor sticker on forearm (left) and measurement with portable VNA with custom two-loop antenna head (right) (note: written informed consent was obtained from participants to use selected photos). Interhuman and intrahuman variation of *f*_o_ (**b**) and ∆TL (**c**) for various regions of the body; population = 4, replicates = 5, error bars show s. d. *x*-direction translation effect on ∆TL (**d**) and *f*_o_ (**g**). *y*-direction translation effect on ∆TL (**e**) and *f*_o_ (**h**). Angle between reader antenna and resonator effect on ∆TL (**f**) and *f*_o_ (**i**); error bars show s. d.

To prepare our sweat sensors for a larger-scale human trial, we first conducted an initial feasibility study with our resonators on a smaller cohort of human subjects to determine what level of sensor variance we can expect from these two confounding factors (local dielectric changes and sensor-reader orientation) (Fig. 5a). These studies investigated both inter- and intra-human variation in TL responses with four male subjects being tested on 4 different areas of the body (forearm, stomach, lower back, thigh, and calf (Fig. 5b, c)). Each body part was scanned five times with the reader replaced on top of the patch each time to account for repositioning effect. The average standard error for *f*_o_ and TL was 156 kHz and 0.08 dB respectively. Given the dynamic range for ∆TL (6 dB) and ∆*f*_o_ (300 kHz) this would mean an expected signal to noise ratio of 75 for ∆TL magnitude and 1.92 for ∆*f*_o_. A high signal to noise ratio (>3) for TL magnitude indicates sweat conductance is not impaired by spatial variation between reader and resonator. However, for sweat rate, the reader sweat sticker orientation creates enough variation in the resonant frequency that it will prove difficult for analysis unless further mitigated.

To further understand the effect of orientation (in *x*,*y*,*z*, and angle dimensions) between the resonator and reader, we conducted a few additional experiments. The reader head (which contains the two looped coils, which inductively couple with the resonator) occupies an area of 9 ×6 cm. The 5 cm diameter sticker was then oriented in the top right corner of the reader head where all analysis was performed for this study and translated in the *x-* and *y-*directions, as well as rotated. The results show major changes (>10 dB) in the *x*-direction and with increasing angle for TL with corresponding changes in frequency also being large with > 4 MHz (Fig. 5d–g). Less severe changes were found in the *y*-direction with changes <1.2 dB for TL and <0.7 MHz for frequency (Fig. 5h, i), however, these deviations are relatively large compared to the dynamic range of the sweat sticker response to different conductivities and distance filled. The *z*-direction was also analyzed with responses very similar to angular changes (see Fig. 3c and Supplementary Fig. 6).

## Discussion

This study presents an initial prototype of a wireless resonant sweat sensor for monitoring undercoat/PPE perspiration. The sensor demonstrates the ability to monitor both sweat conductivity and sweat rate using the same transmission loss parameter. However, limitations still exist that need to be addressed before a human study is conducted to test sweat collection on-body, under PPE or other garments. Although the effect of garment thickness on sweat conductivity response can be corrected through a linear offset relation, the effect of sweat rate does not have a simple correction factor. Instead, the sensor must be calibrated through different thicknesses and types of material before use. Other limitations are highlighted in our initial performance study of resonators placed on the body and scanned after repositioning. This data demonstrates the importance of carefully repositioning the reader and sensor in the same orientation with one another between consecutive measurements, which validates previously known phenomena involving inductively coupled systems^55^. In order to achieve consistent orientation using the portable VNA reader, two possible strategies could be employed. One strategy would be to guide the reader into the same orientation for each scan, such as with mechanical tabs, aligning magnets, or with embedded hall sensors with LED activation when properly aligned. Another strategy could be employing an embedded array of resonant sensors to correct for positional changes^56^. Alternatively, the frequency and TL dynamic range could be increased by changing the proximity of the microfluidic channel with respect to the resonator. The EM fields produced by the resonator weaken rapidly as a function of distance, and the current separation distance of 0.75 mm between the fluid and resonator coil is quite significant considering this is a near field application. This strategy of increasing the sensor signal would provide a more robust mitigation strategy than assuring proper orientation alone. Likely a combination of these strategies will be the most robust to implement before doing a large scale human trial of the sweat analysis sticker. Further work will be done on this front to fabricate the channels closer to the resonator EM fields, as well as engineer mechanisms to assure proper placement of reader on sticker for analysis.

We have demonstrated a passive (no on-board power) wearable sweat rate and sweat conductivity sensor that can be measured wirelessly through clothing or PPE without the need for circuit boards. The sensor has a unique orthogonal response, which transduces the conductivity of the sweat through changes in transmission loss magnitude and sweat rate through rate of change of resonant frequency. In particular, the sweat sensor can robustly transduce the sweat conductivity through any non-metal PPE or clothing when accounting for the thickness of the material. These data were collected using a low-cost (<$500) portable VNA reader. The single-use sensor could be used to determine local sweat loss through multiple scans over perspiration period. The resonant sticker can also be interrogated through thick, opaque PPE with potential applications in monitoring sweat loss and hydration in firefighters during firefighting activities. Further work needs to be done in order to increase the frequency and amplitude response by fabricating the microfluidic change more proximal to the resonator, as well as developing strategies that ensure consistent orientation between resonator and reader. These stickers could also be further designed to selectively monitor biomarkers in sweat (electrolytes, metabolites, and peptides), which would fill a need for a more sensitive sensor to monitor sweat biomarkers in situ.

## Methods

### Resonator fabrication

Copper coated polyimide (DuPont Pyralux) for the flexible planar spiral resonators was purchased from Adafruit (35 µm copper layer, 25 µm polyimide layer). An indelible marker mask in the form of an Archimedean spiral was applied to pyralux using an X–Y plotter (Silhouette Curio). The resonator had an outer diameter of 40 mm and pitch of 1.2 mm and was designed in InkScape (see Supplementary Fig. 7). These dimensions were chosen so that the resonator frequency of the bare resonator would be ~85 MHz, which is below the upper frequency limit (250 MHz) of the portable VNA (MetroVNA). Copper was chemically etched around the mask using H_2_O_2_/HCl solution in a 2:1 volume ratio. Finally, the indelible mask was removed using acetone and air dried. The final resonator was then adhered to a microfluidic chip using acrylic adhesive.

### Microfluidic chip fabrication

PDMS (Dow Sylguard 184) for flexible microfluidic chips was purchased from Ellsworth Adhesives. The microfluidic chips were fabricated via laser ablation using a craft laser cutter (GlowForge Plus). Designs were made in InkScape and saved in a scalable vector graphics format to ensure vector mode operation of the laser cutter. Models for the depth and width of the channels were developed by cutting linear channels into PDMS with varying laser cutter powers and speeds settings between 8 and 24 W and between 0.008 and 0.04 m/s, respectively (Supplementary Table 1). A total of 88 channels were analyzed with 22 different settings with four replicates per setting. The widths and depths of the linear channels were determined with an Olympus DSX110 3D microscope. The data collected was then used to fit linear models for depth and width of laser-ablated channels as a function of cutting speed and laser power. The microfluidic chip made for the sweat analysis sticker was fabricated in PDMS (thickness 1 mm) with using 0.024 m/s cutting speed and 16 W laser power.

The laser-ablated channels were then bonded and sealed with another layer of PDMS (thickness 1 mm) using a plasma bonding process. Both layers of PDMS were treated in a plasma treated for 30 seconds in O_2_ plasma (Plasma Etch PE-25). The pieces were then immediately placed together and bonded using finger pressure followed by a baking period in an oven at 60 °C for 10 min. Bonded channels were than adhered to a resonator using acrylic adhesive (thickness 100 µm) for a total sensor thickness of 2.16 mm.

### Conductive NaCl solutions

NaCl used for all experiments was purchased from Sigma Aldrich. Salt solutions between 0.01 M and 0.1 M as this is the biologically relevant range of normal NaCl concentrations in sweat^46^. Conductivity of the solutions could be calculated by taking the product of the molar conductivity, the concentration, and the activity coefficient of NaCl solution, which can be calculated from the Debye–Huckel limiting law^47^ using values of molar conductivities found in^57^ and complex permittivity^58^. The associated conductivities of the NaCl solutions prepared equaled ~1–10 mS/cm, which was consistent with sweat conductivities reported in the literature^49,59^. These solutions were used as ‘artificial’ sweat solutions to calibrate the sweat sticker to varying conductivities of sweat and to distance fluid traveled in channel.

### Filling channels setup

Channels were filled using a syringe pump (Chemyx Fusion 100) attached to the sticker via tygon tubing (ID 1/8”). In order to visually determine the distance filled indelible ink marks were placed on the microfluidic chip at one-eighth turns at which data points were taken. For both the conductivity and distance filled experiments the chip was filled at one-eighth turn increments. After each fill point, the resonator was interrogated by the VNA as detailed below. For the effect of conductivity on TL the channels were completely filled with conductive solutions between 0.01 and 0.1 mol/L NaCl before each data point was taken.

### Mechanical stability of sensor

The effect of bending on the cross sectional area of the laser-ablated channels was determined by bending cut channels around a curved block of known geometry and taking images of channel cross sections with the Olympus DSX110 digital microscope. Images were subsequently analyzed in MatLab using the Image Segmenter app to determine cross sectional area. A straight edged block and three curved blocks with decreasing radius of curvature (for a sharper bend) were used. For each block five channels were analyzed and average and standard deviation of cross sectional area was calculated.

Cyclic fatigue testing was also performed to determine effect of cyclic bending on sensor performance. The sensor was elastically deformed repeatedly over a curved edge. Before the testing began and after every 500 cycles the frequency shift response was taken by filling the sensor with 0.01 mol/L NaCl until 2000.

### Human study

The human study was conducted with approval and in accordance with the Iowa State University Institutional Review Board. Written informed consent was received from all participants before experiments were performed. Written consent was obtained separately for photographs of subjects used in figures for this study. The population of participants consisted of males ages 18–30 with a BMI under 30. Resonators used for the human study had 40 mm outer diameter and pitch of 1.2 mm with an inner diameter of 10 mm (see Supplementary Fig. 8). Participants arrived to the kinesiology testing facility and were outfitted with resonant sensors. Resonators were attached to the body using Tegaderm (3 M) and were placed on the forearm, stomach, lower back, thigh, and calf of participants. Five scans were taken for each data point to determine the noise of the signal. The average and standard deviations of these scans were calculated and reported from the five resonant sensors on four subjects.

### Reader/resonator orientation study

The effect of *x*, *y*, *z*, and angle orientation of the reader antenna and resonator on the TL output were measured by changing the orientation between the reader and resonator. For the *x-* and *y*-direction this was done by manually moving the resonator across the reader head in either the *x* or *y-*directions between scans. To accurately measure the effect distance displacement a grid printed on paper was taped onto the reader head. For the *z-*direction the resonator was fixed stationary on the lab bench and the reader was attached to a vertical translation stage (Melles Griot) and raised between each scan. The distance raised was measured using scale on the vertical translation stage. For angle orientation the resonator was again fixed stationary on the lab bench and the reader was attached to a rotating lab clamp. The angle between the reader and resonator was increased between each scan with the angle measured using a protractor.

### VNA analysis

A portable VNA (MetroVNA Deluxe 250 MHz) was used for all experiments performed in this study. The VNA was transformed into a resonator reader by connecting it to a custom holder made out of a 3D printed holder with a dual-loop coil antenna. The portable VNA was interfaced with the vnaJ software (vnaj.dl2sba.com) to attain and store data. When excited with RF power from the VNA, the reader antenna generates an electromagnetic field (EM field) that inductively couples with the resonator. The software captures the inphase and quadrature of the RF signal and uses this information to calculate the TL of the device under test, e.g., the resonator and reader antenna. The TL data was analyzed using a script written in MatLab (see Supplementary Note 1), which fit a quadratic model to the peak of the bode plot and then reported the associated TL magnitude and frequency (*f*_o_). To minimize noise five scans were taken and averaged for each measurement.

## Supplementary information

supplementary-materials

reporting-summary

## Data Availability

The data that support the findings of this study are available from the corresponding author upon reasonable request.

## Code availability

Custom code was written in Matlab code version 18b for this study, and has been made available in Supplementary Note 1.
