# Supplementary material for: Sweat monitoring beneath garments using passive, wireless resonant sensors interfaced with laser-ablated microfluidics
Source: NPJ Digit Med. 2020 Apr 30;3:62. doi: 10.1038/s41746-020-0270-2 (PMC7193562; doi:10.1038/s41746-020-0270-2)
Supplement: Supplementary file 1 — supplementary-materials [file 41746_2020_270_MOESM1_ESM.pdf]

Sweat Monitoring Beneath Garments Using Passive, Wireless Resonant Sensors Interfaced with Laser-Ablated Microfluidics

Authors

Adam R. Carr[1], Yash H. Patel[1], Charles R. Neff[1], Sadaf Charkhabi[1], Nathaniel E. Kallmyer[1], Hector F. Angus[2], Nigel F. Reuel[1]*

Affiliations

1. Department of Chemical and Biological Engineering, Iowa State University
2. Department of Kinesiology, Iowa State University

*Corresponding Author – reuel@iastate.edu

Supplementary Materials

Table of Contents

Supplementary Materials 1
Supplementary Figure 1. 1
Supplementary Figure 2. 3
Supplementary Figure 3. 4
Supplementary Figure 4. 4
Supplementary Figure 5. 4
Supplementary Figure 6. 5
Supplementary Figure 7. 6
Supplementary Table 1. 7
Supplementary Figure 8. 8
Supplementary Note 1. 8

Supplementary Figure 1.

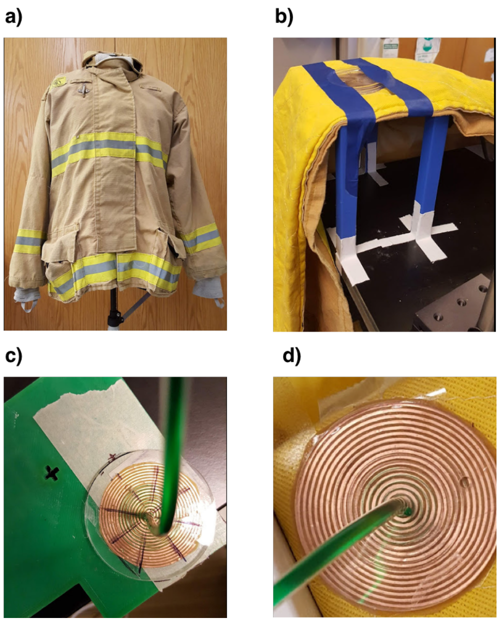

Supplementary Figure 1. a) Morning Pride TAILS PPE, firefighter jacket. b) Stand for reading resonator through PPE. c) Reading resonator directly on reader. d) Reading resonator through PPE.

Supplementary Figure 2.

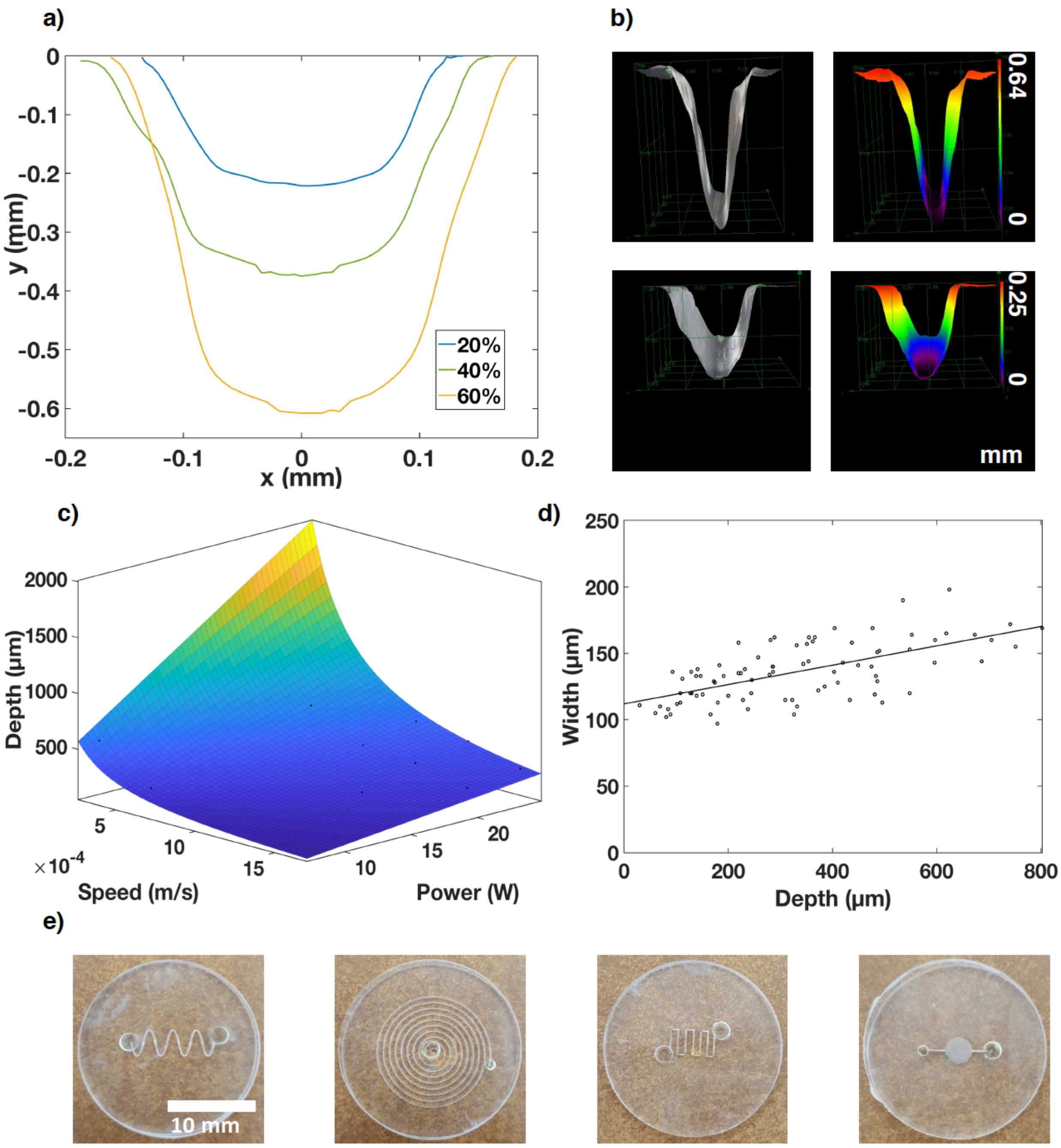

Supplementary Figure 2. Laser ablation of microfluidic channels in PDMS. a) Sample profile data for laser ablated channels from Olympus DSX110 3D microscope. Data was zeroed to the channel top and shown for constant speed of 0.024 m/s with different percent laser power (% 40 W), trials = 22, replicates = 4. b) Sample 3D images of channels with and without contour map. c) Depth model as a function of speed and laser-power. d) Width as a function of depth. e) Designs of microfluidic devices made using laser ablated method, each fabricated within 15 minutes.

Supplementary Figure 3.

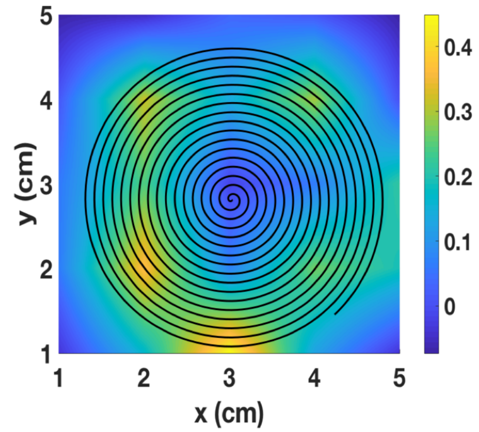

Supplementary Figure 3. Sensitivity of different regions of resonator to changes in dielectric from air to 20 µL of water above that spot. The units of the color bar are in MHz normalized to the frequency response of the resonator exposed to air only.

Supplementary Figure 4.

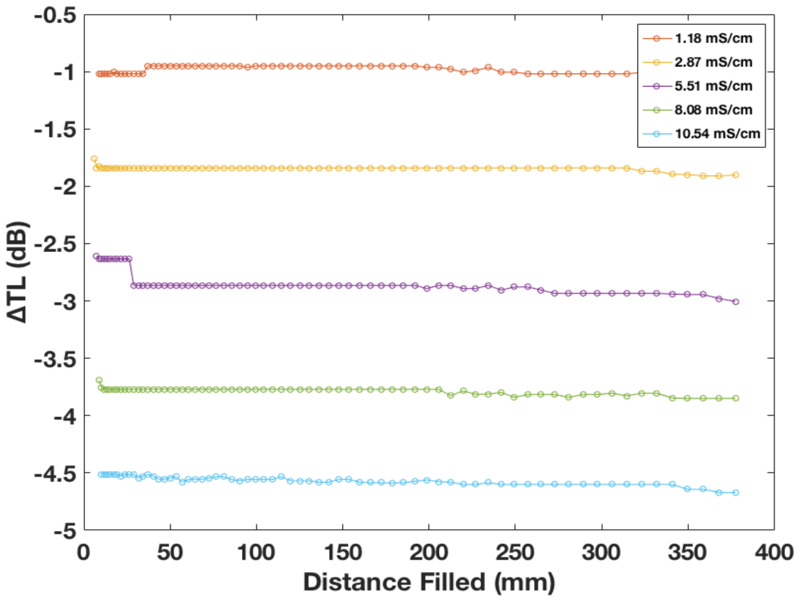

Supplementary Figure 4. Effect of filling on the ΔTL response at different solution conductivities. Note how the effect is negligible between the different solutions.

Supplementary Figure 5.

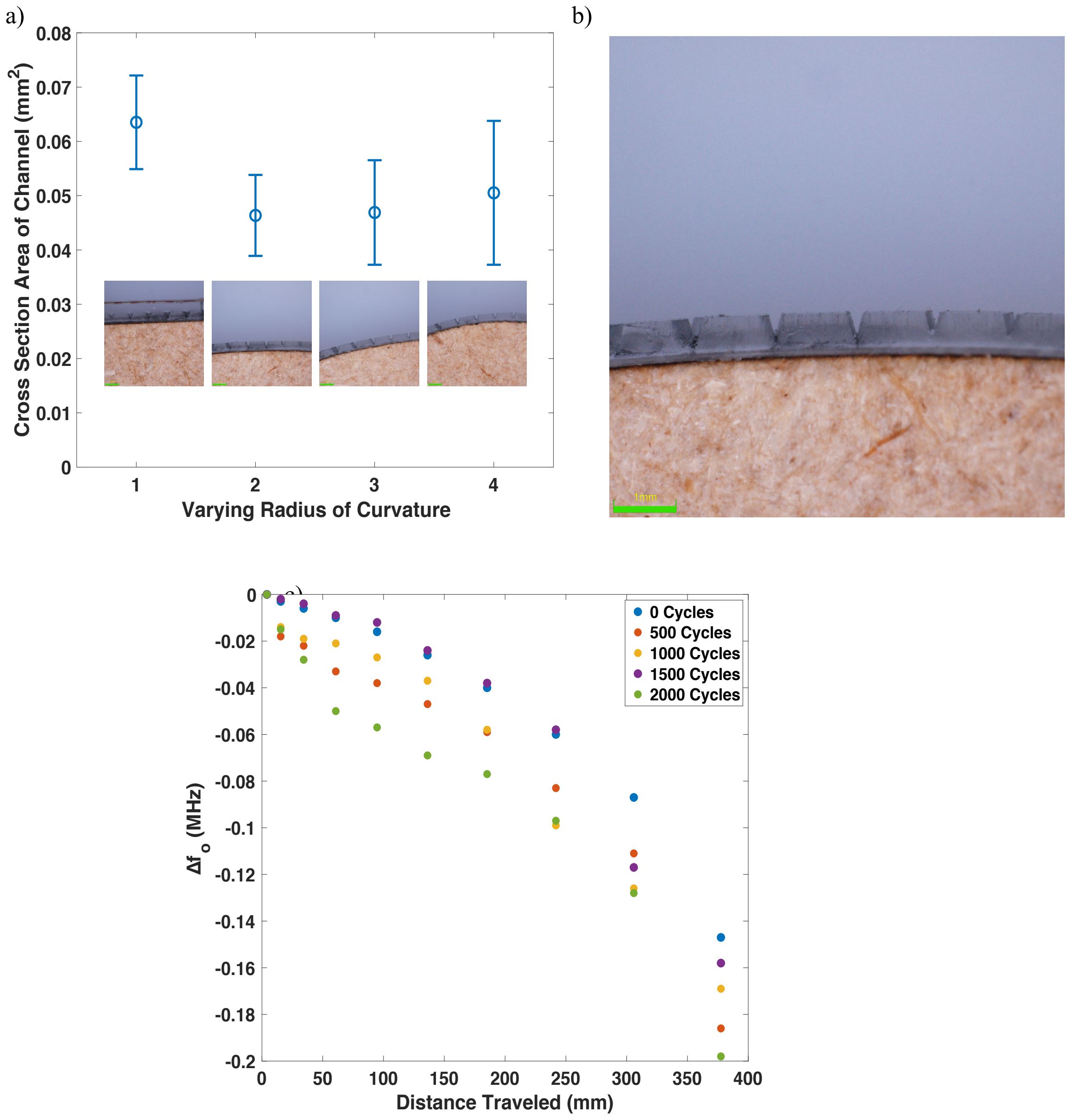

Supplementary Figure 5. a) Effect of bending on the cross sectional area of the PDMS microfluidic channels. 1. being no curvature, 2. large radius of curvature, 3. medium radius of curvature, 4. small radius of curvature.

Supplementary Figure 6.

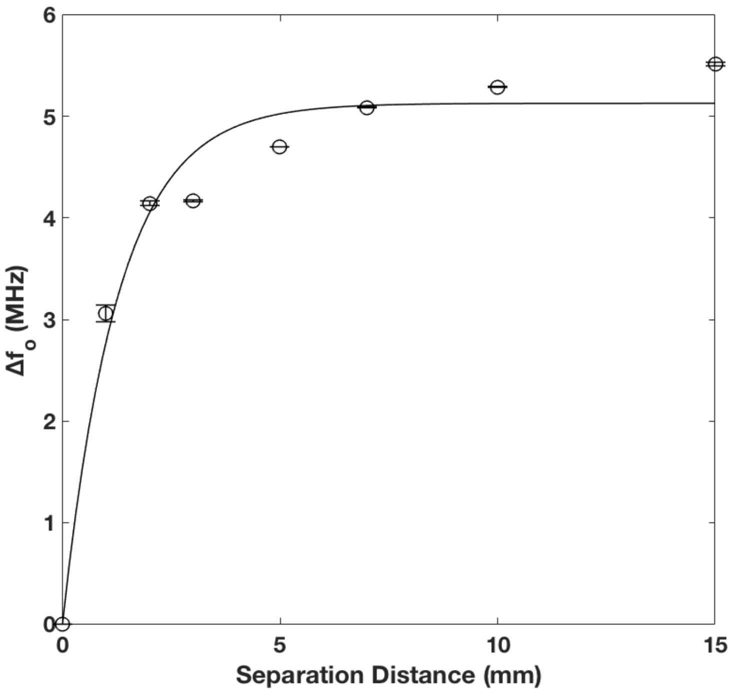

Supplementary Figure 6. Effect of separation distance in the z direction on Δf response.

Supplementary Figure 7.

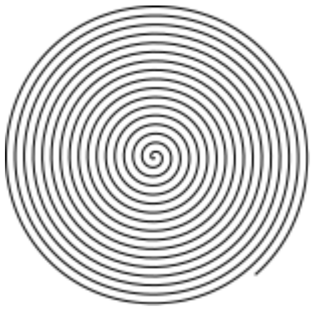

Supplementary Figure 7. InkScape design of resonator used for the sweat analysis sticker. The resonator has an outer diameter of 40 mm and a pitch of 1.2 mm.

Supplementary Table 1.

Supplementary Table 1. Tabulated averaged values for the laser ablated channels used to develop the width and depth models for cutting into PDMS.

speed (m/s)	power (W)	width_avg (mm)	depth_avg (mm)	width_std (mm)	depth_std (mm)
0.000328333	12	0.15875	0.71275	0.012038134	0.033751852
0.000328333	8	0.1645	0.58	0.023923489	0.031622777
0.000655417	12	0.13575	0.3435	0.0156285	0.039277856
0.000655417	8	0.131	0.277	0.010984838	0.036912058
0.000655417	20	0.15325	0.6515	0.023128266	0.118009534
0.000655417	16	0.15	0.451	0.021181753	0.07256032
0.0009825	12	0.1275	0.19525	0.005446712	0.029097895
0.0009825	8	0.12575	0.13275	0.007274384	0.014131083
0.0009825	20	0.161	0.421	0.007438638	0.060708319
0.0009825	16	0.156	0.286	0.008164966	0.047471044
0.0009825	24	0.162	0.511	0.02072036	0.060712437
0.001312083	12	0.11675	0.14425	0.017461863	0.035548383
0.001312083	8	0.10425	0.1035	0.005909033	0.043985793
0.001312083	20	0.1235	0.38875	0.01034408	0.0473623
0.001312083	16	0.11675	0.24975	0.010688779	0.046863499
0.001312083	24	0.1325	0.46175	0.013699148	0.029422568
0.001636667	12	0.1185	0.12075	0.011676187	0.034622067
0.001636667	8	0.114	0.08125	0.007348469	0.038362579
0.001636667	20	0.1335	0.28925	0.016421531	0.050305939
0.001636667	16	0.12875	0.16375	0.013985111	0.043355363
0.001636667	24	0.14	0.33875	0.01197219	0.094882493

Supplementary Figure 8.

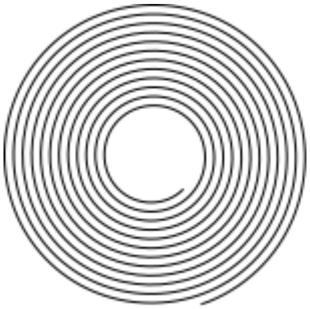

Supplementary Figure 9. InkScape design of resonator used for the human study. The resonator has an outer diameter of 40 mm and a pitch of 1.2 mm and an inner diameter of 10 mm.

Supplementary Note 1.

1. Main File: This code is the main script which calls for user input, such as what parameter to measure (in this study all parameters monitored were TL). User specifies the project folder name, experiment name, the independent variable type, number of points, number of scans, and indices in those scans to determine peak value.

```
%MetroVNA
%Coded by Adam Carr 12.14.2018
%For easier work flow and ability to monitor than one parameter
%from the MetroVNA at a time

%filenames
parameter = input('Put 1 for TL or 0 for RL');
errorbars = input('Error bars 1 for yes 0 for no');
ProjectName = input('Enter Project name: ');%project fileName example
'Hydration Study'
FolderName = input('Enter Folder name: ');%subject fileName example
'Subject1'
indVar = input('Enter independant variable type: ');%min, temp, x-axis
high = input('Enter the max independant variable: ');%how many time points
are there, if 450 files and 5 files/time point then 18 points
samples = input('Number of samples: ');% usually 5, unless freescan
n = high*samples;
startIndex = input('Start index: ');%start searching for max/min
endIndex = input('End index: ');%end searching for max/min

if parameter == 1
    %n =Rename(ProjectName,FolderName); %Rename files in the project folder

    [stdPFTL,PeakFrequencyTL] =
PeakFrequency(n,ProjectName,FolderName,samples,startIndex,endIndex);
    [stdDFTL,DipFrequencyTL] =
DipFrequency(n,ProjectName,FolderName,samples,startIndex,endIndex);
    [stdPGTL,PeakGainTL] =
PeakLoss(n,ProjectName,FolderName,samples,startIndex,endIndex);
    [stdDGTL,DipGainTL] =
DipLoss(n,ProjectName,FolderName,samples,startIndex,endIndex);

    if errorbars == 0
        x = linspace(0,high,n/samples);
        A(:,1) = x;
```

```
        A(:,2) = PeakFrequencyTL;
        A(:,3) = DipFrequencyTL;
        A(:,4) = PeakGainTL;
        A(:,5) = DipGainTL;
        A(:,6) = stdPFTL;
        A(:,7) = stdDFTL;
        A(:,8) = stdPGTL;
        A(:,9) = stdDGTL;

        oldFolder = (['/Users/adamcarr/Box Sync/Macbook Pro/MATLAB/VNA
Data']);
        newFolder = (['/Users/adamcarr/Box Sync/Macbook Pro/MATLAB/VNA
Data/',ProjectName,'/',FolderName]);
        cd(newFolder);
        csvwrite('comparison.csv',A);
        figure(1)
        one = plot(x,PeakFrequencyTL,'ro');
        saveas(one,'Peak_Frequency.png')
        xlabel(indVar)
        ylabel('Peak Frequency (MHz)')
        set(gca,'FontSize',16,'fontWeight','bold')
        set(findall(gcf,'type','text'),'FontSize',16,'fontWeight','bold')
        figure(2)
        two = plot(x,DipFrequencyTL,'bo');
        saveas(two,'Dip_Frequency.png')
        xlabel(indVar)
        ylabel('Dip Frequency (MHz)')
        set(gca,'FontSize',16,'fontWeight','bold')
        set(findall(gcf,'type','text'),'FontSize',16,'fontWeight','bold')
        figure(3)
        three = plot(x,PeakGainTL,'ko');
        saveas(three,'Peak_Gain.png')
        xlabel(indVar)
        ylabel('Peak TL (dB)')
        set(gca,'FontSize',16,'fontWeight','bold')
        set(findall(gcf,'type','text'),'FontSize',16,'fontWeight','bold')
        figure(4)
        four = plot(x,DipGainTL,'ko');
        saveas(four,'Dip_Gain.png')
        xlabel(indVar)
        ylabel('Dip TL (dB)')
        set(gca,'FontSize',16,'fontWeight','bold')
        set(findall(gcf,'type','text'),'FontSize',16,'fontWeight','bold')
        cd(oldFolder);
    end

    if errorbars == 1
        x = linspace(0,high,n/samples);
        A(:,1) = x;
        A(:,2) = PeakFrequencyTL;
        A(:,3) = DipFrequencyTL;
        A(:,4) = PeakGainTL;
        A(:,5) = DipGainTL;
        A(:,6) = stdPFTL;
        A(:,7) = stdDFTL;
        A(:,8) = stdPGTL;
        A(:,9) = stdDGTL;
        oldFolder = (['/Users/adamcarr/Box Sync/Macbook Pro/MATLAB/VNA
Data']);
        newFolder = (['/Users/adamcarr/Box Sync/Macbook Pro/MATLAB/VNA
Data/',ProjectName,'/',FolderName]);
        cd(newFolder);
        csvwrite('comparison.csv',A);
```

```
        figure(1)
        one = errorbar(x,PeakFrequencyTL,stdPFTL,'ro');
        saveas(one,'Peak_Frequency.png')
        xlabel(indVar)
        ylabel('Peak Frequency (MHz)')
        set(gca,'FontSize',16,'fontWeight','bold')
        set(findall(gcf,'type','text'),'FontSize',16,'fontWeight','bold')
        figure(2)
        two = errorbar(x,DipFrequencyTL,stdDFTL,'bo');
        saveas(two,'Dip_Frequency.png')
        xlabel(indVar)
        ylabel('Dip Frequency (MHz)')
        set(gca,'FontSize',16,'fontWeight','bold')
        set(findall(gcf,'type','text'),'FontSize',16,'fontWeight','bold')
        figure(3)
        three = errorbar(x,PeakGainTL,stdPGTL,'ko');
        saveas(three,'Peak_Gain.png')
        xlabel(indVar)
        ylabel('Peak TL (dB)')
        set(gca,'FontSize',16,'fontWeight','bold')
        set(findall(gcf,'type','text'),'FontSize',16,'fontWeight','bold')
        figure(4)
        four = errorbar(x,DipGainTL,stdDGTL,'ko');
        saveas(four,'Dip_Gain.png')
        xlabel(indVar)
        ylabel('Dip TL (dB)')
        set(gca,'FontSize',16,'fontWeight','bold')
        set(findall(gcf,'type','text'),'FontSize',16,'fontWeight','bold')
        cd(oldFolder);
    end
end

if parameter == 0
   % n = Rename(ProjectName,FolderName); %Rename files in the project
folder

    [stdDFTL,DipFrequencyTL] =
DipFrequency(n,ProjectName,FolderName,samples,startIndex,endIndex);
    [stdDGTL,DipGainTL] =
DipLoss(n,ProjectName,FolderName,samples,startIndex,endIndex);

    if errorbars == 0
        x = linspace(0,high,n/samples);
        A(:,1) = x;
        A(:,2) = DipFrequencyTL;
        A(:,3) = DipGainTL;
        A(:,4) = stdDFTL;
        A(:,5) = stdDGTL;
        oldFolder = (['/Users/adamcarr/Box Sync/Macbook Pro/MATLAB/VNA
Data']);
        newFolder = (['/Users/adamcarr/Box Sync/Macbook Pro/MATLAB/VNA
Data/',ProjectName,'/',FolderName]);
        cd(newFolder);
        csvwrite('comparison.csv',A);
        cd(oldFolder);
        figure(1)
        plot(x,DipFrequencyTL,'bo');
        xlabel(indVar)
        ylabel('S11 Frequency (MHz)')
        set(gca,'FontSize',16,'fontWeight','bold')
        set(findall(gcf,'type','text'),'FontSize',16,'fontWeight','bold')
        figure(2)
        plot(x,DipGainTL,'ko');
```

```
        xlabel(indVar)
        ylabel('TL (dB)')
        set(gca,'FontSize',16,'fontWeight','bold')
        set(findall(gcf,'type','text'),'FontSize',16,'fontWeight','bold')
    end

    if errorbars == 1
        x = linspace(0,high,n/samples);
        A(:,1) = x;
        A(:,2) = DipFrequencyTL;
        A(:,3) = DipGainTL;
        A(:,4) = stdDFTL;
        A(:,5) = stdDGTL;

        oldFolder = (['/Users/adamcarr/Box Sync/Macbook Pro/MATLAB/VNA
Data']);
        newFolder = (['/Users/adamcarr/Box Sync/Macbook Pro/MATLAB/VNA
Data/',ProjectName,'/',FolderName]);
        cd(newFolder);
        csvwrite('comparison.csv',A);
        cd(oldFolder);
        figure(1)
        errorbar(x,DipFrequencyTL,stdDFTL,'bo');
        xlabel(indVar)
        ylabel('S11 Frequency (MHz)')
        set(gca,'FontSize',16,'fontWeight','bold')
        set(findall(gcf,'type','text'),'FontSize',16,'fontWeight','bold')
        figure(2)
        errorbar(x,DipGainTL,stdDGTL,'ko');
        xlabel(indVar)
        ylabel('TL (dB)')
        set(gca,'FontSize',16,'fontWeight','bold')
        set(findall(gcf,'type','text'),'FontSize',16,'fontWeight','bold')
    end
end

set(gca,'FontSize',16,'fontWeight','bold')
set(findall(gcf,'type','text'),'FontSize',16,'fontWeight','bold')

end
```

2. Peak Frequency: This function determines the peak frequency of the VNA scan by fitting a quadratic to the points around the maximum point.

```
function [stdAvg,PeakFreq1avg] =
PeakFrequency(n,ProjectName,FolderName,samples,startIndex,totSamples)
% Coded by NFR on 8.29.2018
%modified by Sadaf on 10.14.2018 to plot all single S21 vs Freq and
%modified by Adam on 3.14.2019
%identify the peak spot
% This code plots the dynamic response of data acquired by the portable VNA
%
close all;
oldFolder = (['/Users/adamcarr/Box Sync/Macbook Pro/MATLAB/VNA Data']);
newFolder = (['/Users/adamcarr/Box Sync/Macbook Pro/MATLAB/VNA
Data/',ProjectName,'/',FolderName]);
cd(newFolder);

PeakFreq1 = zeros(1,n);

for i = 1:n
    D = csvread([num2str(i),'.csv'],1);
```

```
        X = D(startIndex:totSamples,1)./10^6;
        Y = D(startIndex:totSamples,2);

        [Y2,posY2] = findpeaks(Y); %Find all the potential peak and record
    vector location

        [Peak1,posPeak1] = max(Y2); %Find max peak and record the vector
    location
         % V4 use quadratic fit around local points
         pos3 = posY2(posPeak1); % This is the index of the max peak in the
    original vector.
         nq = 10;
         if pos3-5<1
             % The VNA collected strange data on this step, use last PeakFreq
             PeakFreq1(i) = Freqloc;

         else
             Xloc = X(pos3-nq:pos3+nq,1);
             Yloc = Y(pos3-nq:pos3+nq,1);
             p = polyfit(Xloc,Yloc,2);
             Freqloc = -p(2)/(2*p(1));
             Magloc = (p(1)*Freqloc*Freqloc)+(p(2)*Freqloc)+p(3);
             M = 10;
             PeakFreq1(i) = Freqloc;
         end
    end

    % samples = 5;
    % % PeakFreq1 = reshape(cumsum(ones(samples,10),2),[],1);
    % PeakFreq1avg = arrayfun(@(i) mean(PeakFreq1(i:i+samples-
    1)),1:samples:length(PeakFreq1) - samples + 1);

    % samples = 5;
    PeakFreq1avg = arrayfun(@(i) mean(PeakFreq1(i:i+samples-
    1)),1:samples:length(PeakFreq1) - samples + 1);
    stdAvg = arrayfun(@(i) std(PeakFreq1(i:i+samples-
    1)),1:samples:length(PeakFreq1) - samples + 1);



    cd(oldFolder);
    end
```

3. Peak TL: This function determines the magnitude of the peak value in similar fashion to the above function.

```
function [stdAvg,lossAvg] =
PeakLoss(n,ProjectName,FolderName,samples,startIndex,totSamples)

close all;
oldFolder = (['/Users/adamcarr/Box Sync/Macbook Pro/MATLAB/VNA Data']);
newFolder = (['/Users/adamcarr/Box Sync/Macbook Pro/MATLAB/VNA
Data/',ProjectName,'/',FolderName]);
cd(newFolder);

loss = zeros(1,n);

for i = 1:n
    D = csvread([num2str(i),'.csv'],1);
    X = D(startIndex:totSamples,1)./10^6;
    Y = D(startIndex:totSamples,2);

    [Ymin2,~] = findpeaks(Y);
```

```
    [PeakMin,~] = max(Ymin2); %Find min peak and record the vector location
    loss(i) = PeakMin;

end
% csvwrite('signalLoss.csv',loss)

% samples = 5;
lossAvg = arrayfun(@(i) mean(loss(i:i+samples-1)),1:samples:length(loss) -
samples + 1);
stdAvg = arrayfun(@(i) std(loss(i:i+samples-1)),1:samples:length(loss) -
samples + 1);

cd(oldFolder);
end
```
